# Enrichment-free analysis of anionic micropollutants in the sub-ppb range in drinking water by capillary electrophoresis-high resolution mass spectrometry

**DOI:** 10.1007/s00216-020-02525-8

**Published:** 2020-03-09

**Authors:** Oliver Höcker, Tobias Bader, Torsten C. Schmidt, Wolfgang Schulz, Christian Neusüß

**Affiliations:** 1 Department of Chemistry, Aalen University, Beethovenstraße 1, 73430 Aalen, Germany; 2 Instrumental Analytical Chemistry and Centre for Water and Environmental Research (ZWU), University of Duisburg-Essen, Universitaetsstrasse 5, 45141 Essen, Germany; 3 Laboratory for Operation Control and Research, Zweckverband Landeswasserversorgung, Am Spitzigen Berg 1, 89129 Langenau, Germany; 4 IWW Zentrum Wasser, Moritzstrasse 26, 45476 Mülheim an der Ruhr, Germany

**Keywords:** Capillary electrophoresis/electrophoresis, Mass spectrometry, Water analysis, Interfacing, Micropollutants, Persistent and mobile organic contaminants

## Abstract

Reversed-phase liquid chromatography (RPLC) used for water analysis is not ideal for the analysis of highly polar and ionic contaminants because of low retention. Capillary electrophoresis (CE), on the other hand, is perfectly suited for the separation of ionic compounds but rarely applied in environmental analysis due to the weak concentration sensitivity when coupled to mass spectrometry (MS). However, novel interface designs and MS technology strongly improve the sensitivity. Here, a method is presented enabling the screening of anionic micropollutants in drinking water without sample pretreatment by coupling of CE to an Orbitrap mass spectrometer by a nanoflow sheath liquid interface. Targeted analysis of halogenated acetic acids, trifluoromethanesulfonic acid, and perfluorooctanoic and perfluorooctanesulfonic acid was conducted in drinking water samples which were chlorinated for disinfection. A bare fused silica capillary with an optimized background electrolyte (BGE) for separation consisting of 10% acetic acid with 10% isopropanol with large volume sample injection and optimized interface parameters offer limits of quantification in the range of < 0.1 to 0.5 μg/L with good linearity (*R*^2^ > 0.993) and repeatability (14% standard deviation in area). Concentrations of the target analytes ranged from 0.1 to 6.2 μg/L in the water samples. Masses corresponding to halogenated methanesulfonic acids have been found as suspects and were subsequently verified by standards. Mono-, dichloro-, and bromochloro methanesulfonic acid were quantified in a range of 0.2 to 3.6 μg/L. Furthermore, five sulfonic acids, four organosulfates, and the artificial sweeteners acesulfame and cyclamate as well as inorganics such as halides, halogenates, phosphate, and sulfate could be determined as suspects among more than 300 features in a non-targeted screening. Overall, this approach demonstrates the great potential of CE-nanoESI-MS for the screening of ionic contaminants in environmental samples, complementary to chromatographic approaches.

**Electronic supplementary material:**

The online version of this article (10.1007/s00216-020-02525-8) contains supplementary material, which is available to authorized users.

## Introduction

The monitoring of persistent mobile organic contaminants (PMOCS) in the aquatic environment is difficult because conventional reversed-phase chromatographic approaches exhibit low retention. The number of these highly polar or ionic substances in the aquatic environment is constantly increasing due to anthropogenic activity, but the monitoring is only possible to a limited extent. This is a threat for the water resources, because it is expected that many PMOCs cannot be removed by wastewater treatment or drinking water production plants. In addition, disinfection agents like chlorine, ozone, and others, used for the reduction of pathogenic microorganisms, can react with all kinds of natural and anthropogenic substances to a vast amount of more polar/ionic molecules. And while many of these disinfection byproducts (DBPs) have been identified, only few are regulated and it is unknown how many PMOCs are still hidden [1, 2]. Since conventional reversed-phase liquid chromatography (RPLC) and GC separation mechanisms depend on nonpolar interaction, the analytical repertoire is extended by mixed-mode materials, hydrophobic interaction chromatography (HILIC) or ion chromatography (IC) in combination with mass spectrometry [3, 4]. E.g., mixed-mode solid-phase extraction for analyte enrichment in combination with a mixed-mode separation column enabled the fast analysis of 23 target PMOCs with good separation in surface and drinking water, reaching limits of detection (LOD) below 50 ng/L [5–7]. Mixed-mode columns offer the advantage of combining reversed-phase and ion exchange properties for the separation of highly polar substances and can be used in widely available HPLC systems. The analysis of glyphosate and AMPA in drinking water by CE-MS was shown, reaching limits of quantification (LOQ) of low micrograms per liter without derivatization in high ionic strength matrices [8]. In a non-targeted HILIC-MS approach, halogenated methanesulfonic acids (HMSAs) were discovered recently by Zahn et al. [9, 10] for the first time in surface and drinking water. This class of new emerging DBPs was tentatively discovered and later confirmed by standards in subsequent analyses in concentrations between 0.07 and 11.5 μg/L. HILIC-MS is becoming more popular because it extends the range of polarity compared to reversed phase, but for aqueous samples, a solvent exchange is necessary to enable sufficient retention, and often, SPE is needed for sample preparation. While SPE is useful for preconcentration, there is always a risk of losing analytes in this step, which can be problematic for non-targeted approaches to discover new analytes of concern.

Capillary electrophoresis is perfectly suited to separate ionic analytes in aqueous samples due to the high selectivity and complementary separation mechanism to RPLC. Studies have found that capillary electrophoresis might be complementary to HILIC in finding analytes and therefore useful to extend the range of charged analytes due to its separation mechanism based on the charge-to-size ratio [11]. However, CE was rarely applied for the analysis of pollutants in water analysis [12–14], which is mostly due to the weak concentration sensitivity. To some extent, this can be compensated by online preconcentration techniques such as inline-SPE [15] or stacking [16]. CE-MS was applied for the analysis of halogenated acetic acids (HAAs) in water samples with an acetic acid and methanol-based electrolyte [17]. Detection limits between 1600 and 100 μg/L (*S*/*N* = 3) could be achieved by direct analysis with a sheath liquid containing trimethylamine, while a preconcentration with SPE of 30 mL water could lower the LODs to 0.3–7.6 μg/L. Zhang et al. [18] published a method using a sophisticated online preconcentration by balancing the electroosmotic flow by external pressure during injection with enhancement factors of over 3500 over hydrodynamic injection, reaching LODs of 0.013 to 0.12 μg/L with a standard CE-MS interface. This demonstrates the suitability of CE-MS for drinking water analysis applying efficient online preconcentration due to the low conductivity of such samples. With modern LC-MS and mixed-mode solid-phase extraction (SPE) preconcentration, Hu et al. [19] showed a method for the quantitation especially for iodinated HAAs with LODs between 0.02 and 0.48 ng/L.

For perfluorinated alkylated substances (PFAs), a CE method with UV detection and SPE preconcentration reports LODs in the low nanogram-per-liter range with a non-aqueous buffer system and detectable concentrations of 0.8 ng/L in a spiked river sample [20]. Even though high-resolution electrospray mass spectrometry (HRMS) offers great possibilities for identification, the coupling of CE with conventional (sheath liquid) interfaces limits the sensitivity. High sheath liquid flow rates in standard interfaces (1 to 10 μL/min), compared to the low nanoliter-per-minute flow CE effluent, highly dilute the sample zone leading to limited sensitivity. In recent years, significant progress has been made in order to increase the sensitivity of CE-MS coupling by development of new emitter designs, reduction of the sheath liquid flow rate, or even direct spray of the background electrolyte [12]. These nanoflow sheath liquid or sheathless interfaces are characterized by the use of spray tips with openings in the micrometer range and liquid flows far below 1 μL/min, thus reducing analyte dilution. Furthermore, these interfaces produce smaller initial droplet sizes which is beneficial for gas-phase ion production and MS sampling. Enhancement of sensitivity by 30 to 100 times was reported in many applications such as protein analysis [21] and metabolomics [22] and also for small molecules like sulfonic acids [23], relevant for water analysis. However, to the best of our knowledge, there is no report on the direct screening (without SPE preconcentration) of ionic micropollutants in the sub-microgram per liter range, as required for most cases when drinking water is analyzed.

Here, we present a method for the separation and quantification of selected anionic contaminants (HAAs, PFAs, and HMSAs) in drinking water by CE-HRMS. The method uses a nanoflow sheath liquid interface, simple large-volume injection, and acidic pH. The target compounds were quantified in seven samples from four drinking water production plants in Germany using chlorination for disinfection purpose. Furthermore, the data were screened for known and unknown anionic environmental contaminants to evaluate the general ability for the analysis and discovery of PMOCs in water samples.

## Materials and methods

### Chemicals and samples

Isopropanol (LC-MS grade), acetic acid, sodium hydroxide, and hydrochloric acid were obtained from Carl Roth GmbH und Co. KG (Karlsruhe, Germany). All solutions were prepared using ultrapure water (18 MΩ*cm at 25 °C, SG Ultra Clear UV from Siemens Water Technologies, USA). Hydrofluoric acid 40% (v/v) was purchased from Merck (Darmstadt, Germany). Dithiothreitol (DTT) and sodium bicarbonate were obtained from Sigma-Aldrich (Steinheim, Germany). “ES Tuning mix” solution was obtained from Agilent Technologies (Palo Alto, CA, USA). Standards of haloacetic acids and PFAs were purchased from different suppliers: dibromochloro-, dichloro-, trichloro-, and bromochloro acetic acid from Sigma-Aldrich; dibromo acetic acid and perfluorooctanoic and perfluorooctanesulfonic acid from Dr. Ehrenstorfer; and trifluoro acetic acid from Alfa Aesar. The HMSA standards chloro-, bromo-, dichloro-, and bromochloromethanesulfonic acid were kindly provided by Daniel Zahn (Fresenius University of Applied Sciences, Idstein, Germany). For external calibration, the standards were solved in ultrapure water. Seven water samples were provided by four different water treatment plants from Germany which were disinfected by chlorination. Samples with the same numbers originate from the same drinking water production plant. Some of the samples were neutralized by addition of thiosulfate which is indicated by the letter “T,” and an additional chlorination step by “C.” The water suppliers agreed that the results are published in an anonymous form.

### Nanoflow CE-MS interface

**Fig. 1:**
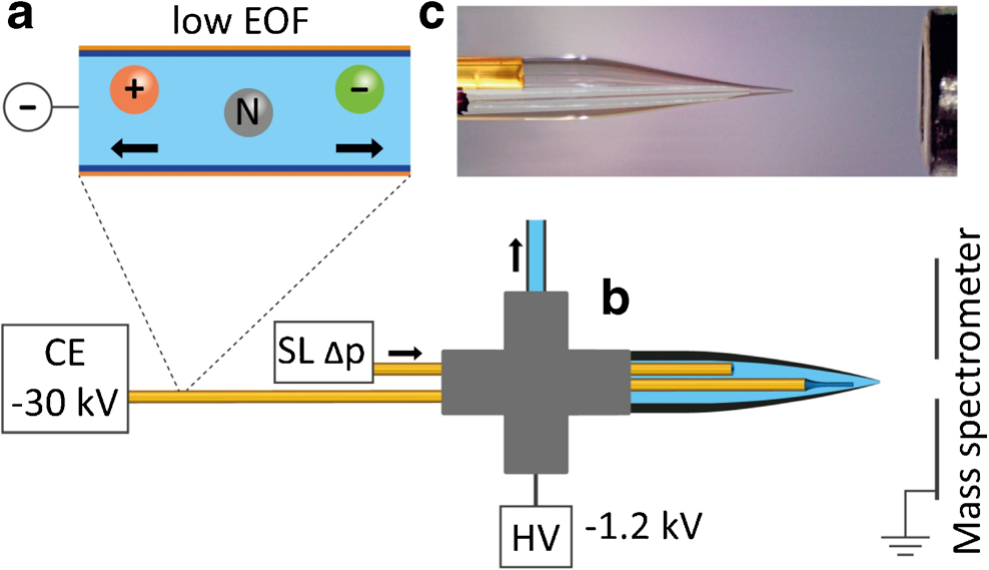
Setup of the nanoflow sheath liquid CE-MS interface for the analysis of strongly acidic compounds in drinking water. (A) 10% (v/v) acetic acid exhibits a low pH (~ 2.2) which causes a low electroosmotic flow enabling negative CE polarity. The separation capillary and a capillary to deliver sheath liquid are guided through a PEEK cross (B) into the borosilicate glass emitter (C). The third arm drains excess liquid and opens the emitter to ambient pressure while the fourth arm is used to ground the CE current and apply the electrospray voltage

The setup of the nanoflow interface is shown in Fig. 1. The separation capillary (50 μm ID, 365 μm OD) is threaded through a PEEK cross-union into a borosilicate electrospray emitter with a 15-μm opening (5.5 cm length, 1.0 mm OD, and 0.75 μm ID). An XYZ-stage allowed positioning of the emitter in front of the MS entrance. The capillary outlet OD was reduced by etching with hydrofluoric acid in the last part (ca. 1 cm) to minimize dead volume inside the emitter tip. The sheath liquid is delivered by a second capillary with 100 μm ID and a blunt tip which is also guided through the cross-union into the emitter, parallel to the separation capillary. A syringe pump delivers sheath liquid continuously, in which the third port of the cross-union is used to drain the excess liquid. Both capillaries can be adjusted in axial position: During analysis, the separation capillary is in frontal position while fresh sheath liquid is delivered some millimeters behind by the second capillary which reduces the carryover of contaminants from the system. In-between analyses, the separation capillary is positioned behind the SL capillary, so that all excess electrolyte and residues from the previous analysis are flushed out backwards and no contamination reaches the emitter tip. The remaining port is used to ground the separation current and to apply the electrospray voltage.

### CE-MS conditions

CE experiments were performed using an Agilent 7100 (Agilent Technologies) coupled to a Thermo Orbitrap Fusion Lumos mass spectrometer (Thermo Fisher Scientific). The separation was conducted in a bare fused silica capillary with 50 μm ID and 60 cm length, initially conditioned with 5 M sodium hydroxide for 15 min, followed by water for 10 min and electrolyte for 30 min. The final electrolyte consisted of 10% acetic acid (v/v) with 10% (v/v) isopropanol. For separation, a potential of − 30 kV was applied to the capillary inlet. Samples were injected hydrodynamically with 100 mbar for 80 s. The sheath liquid consisted of isopropanol:water (50:50 v/v) with 0.5% (v/v) formic acid. A distance of 3.0 mm between the emitter tip and transfer capillary was chosen, giving the highest signal intensities. The Orbitrap mass spectrometer was operated in negative ion mode with 1200 V electrospray potential, full scan from 75 to 510 *m*/*z*, 30,000 resolving power, 64 ms accumulation time, 1E5 AGC target, 45% RF lens, and 4 microscans. MS/MS experiments were conducted with quadrupole isolation in a 1.6-Da window, fragmentation by high collision dissociation (stepped 15, 30, and 45%), and Orbitrap detection with first mass 50 *m*/*z*, AGC target 1E4, and 54 ms maximum injection time.

## Results and discussion

The initial objective of this work was to develop a sensitive method for the separation and identification of HAAs, PFAs, and trifluoromethanesulfonic acid in drinking water samples, ideally avoiding any preprocessing for enrichment. HAAs have pka values between 0.7 and 3.5; thus, BGEs at low pH values were tested for best separation: At low pH, a high selectivity for these HAAs is expected because of a differing degree in deprotonation and resulting net charge. Furthermore, increasing the electrolytes’ pH would result in an EOF towards the capillary inlet, resulting in an interfering flow of sheath liquid into the end of the separation capillary. Avoiding such an EOF would require a coating for EOF suppression. Thus, a fused silica capillary under low pH conditions is preferred since it is inexpensive and can be regenerated easily. Ten percent acetic acid (pH 2.2) and 0.2 (pH 2.2) and 0.5 M formic acid (pH 2.2) were tested as BGE. Formic acid-based BGE showed similar separation but broader peaks than the acetic acid-based BGE. Different organic modifiers such as ethanol, acetonitrile, and isopropanol in concentrations of up to 30% showed changes in selectivity and partially better resolution. However, higher concentration of organic modifiers led to longer separation times and increased peak widths (data not shown). A concentration of 10% acetic acid (v/v) with 10% isopropanol (v/v) was the best compromise between resolution, peak shape, and analysis time and was chosen for final analyses. The samples exhibit a low conductivity compared to the separation electrolyte; thus, a simple and reliable field amplified sample stacking could be used for online preconcentration. Therefore, it was possible to inject up to 20% of the capillary volume (0.24 μL) while preserving sharp bands of around six seconds for the HAAs. The sheath liquid composition turned out to be crucial to achieve the low quantification limits as well as the distance of the emitter tip to the mass spectrometer’s inlet and electrospray voltage. While previous publications [17, 18] tested only alkaline additives, in our case, the addition of formic acid offered significantly higher signal intensities compared to ammonia or volatile ammonium salts. ES voltage and distance were optimized for different sheath liquid compositions by filling the capillary with low concentrated analytes solved in background electrolyte and applying separation voltage while observing signal intensities of selected ion traces. The optimum electrospray voltage of 1200 V, emitter distance of 3.0 mm, and sheath liquid composition of isopropanol:water (50:50 v/v) with 0.5% (v/v) formic acid were confirmed by analysis of injected sample plugs under normal separation conditions. Compared to single capillaries used in setups with similar emitters [21], the interface setup with two capillaries shows improved robustness in overall handling and operation, since particles can be flushed out backwards, which prevents blocking of the tip. When correctly handled, the lifetime of the emitter is several days of continuous analysis. The emitter can be exchanged within several minutes, and after flushing the system for ten minutes, the next analysis can be performed. After emitter change, the performance was evaluated with known analyte standards to ensure reproducible performance.

**Fig. 2:**
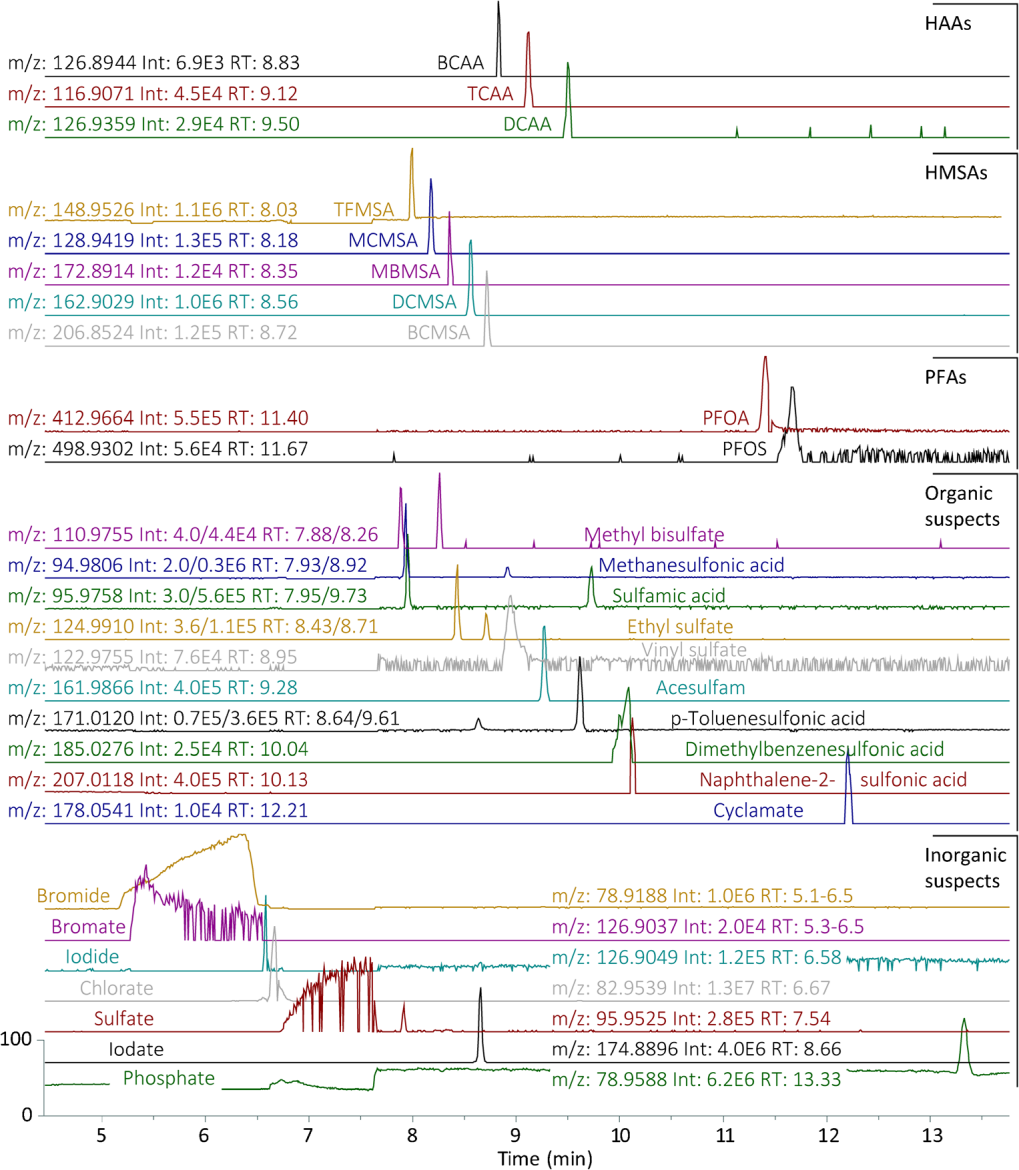
Extracted ion electropherograms (exact mass ± 2.5 ppm) of targeted (HAAs, HMSAs, PFAs) and suspect-targeted analytes in water sample WS-4-C. Signals are normalized due to large variations in signal intensities. Target analytes are confirmed by standard addition, while organic and inorganic suspects are proposals on basis on exact mass sum formula generation (cp. text for details)

Linear calibration curves were derived from six standard concentration levels with triple injection from 10 to 0.1 μg/L of five HAAs (DBCAA, DBAA, DCAA, BCAA, and TCAA) and two fluorosurfactants (PFOS and PFOA) (Table 1). This set-up was used for external calibration and to determine the concentrations in seven drinking water samples from four water suppliers, which were chlorinated for disinfection purposes. The calibration showed a good linearity over all concentrations (*R*^2^ > 0.993, except PFOS with 0.985). Limits of quantification (LOQ) were determined by a signal to noise ratio of 10, if the extracted ion electropherogram (± 2.5 ppm) showed noise. Since this was only the case for three of the substances, the other LOQs were estimated by the lowest point of the calibration curve, where the signal could be detected repeatedly. In accordance with the literature, for most HAAs, the decarboxylated ion species showed higher signal intensity and were chosen for quantitation [24]. The samples were analyzed randomly in triplicates alternating with the calibration standards. Degradation of the analytes in low concentrated standard solutions was observed. Especially the brominated HAAs showed degradation behavior in solution as it was reported previously; hence, the dilutions were prepared right before measurements [25]. Analytes were baseline separated with an average base width of 6 s which indicates a good stacking mechanism. Further, no peak broadening was caused by the (low) dead volume in the transition between the capillary outlet and the emitter tip. Migration times in real samples were shifted by up to 2 min compared to the standards solved in ultrapure water, which is an expected effect for stacking techniques and is caused by differences in total ion concentration of different matrix constituents. To approach this effect, the addition of internal standards would allow the correction of migration time shifts as already used in metabolomics profiling. Here, the identification of the target analytes was confirmed by standard addition. An exemplary separation of BCAA, TCAA, and DCAA in a drinking water sample is shown in Fig. 2.

**Table 1:** Calibration parameters for the determination of concentrations of HAAs, PFAs, and HMSAs in drinking water. LOQs determined by lowest point of calibration curve that could be detected repeatedly (marked by^x^), or signal to noise ratio of 10 if the extracted ion electropherogram (± 2.5 ppm) showed noise (marked by^y^)

Group	Compound	Abbreviation	Formula	Ion	*m*/*z*	Migration time [min ± stdev]	Slope [area*L/μg]	Intercept [area]	*R*^2^	Peak area stdev [%]	LOQ [μg/L]
HAA	Bromochloro acetic acid	BCAA	C_2_H_2_BrClO_2_	[M-COOH]^−^	126.8945	8.6 ± 1.0	233,375	− 35,597	0.997	19	0.1^x^
	Trichloro acetic acid	TCAA	C_2_HCl_3_O_2_	[M-COOH]^−^	116.9060	9.0 ± 1.2	222,671	− 24,312	0.996	11	0.1^x^
	Dichloro acetic acid	DCAA	C_2_H_2_Cl_2_O_2_	[M-H]^−^	126.9348	9.2 ± 0.9	803,858	− 72,020	0.997	11	0.1^x^
	Dibromo acetic acid	DBAA	C_2_H_2_Br_2_O_2_	[M-COOH]^−^	170.8440	9.9 ± 0.8	609,153	− 56,276	0.997	15	0.1^x^
	Dibromochloro acetic acid	DBCAA	C_2_HBr_2_ClO_2_	[M-COOH]^−^	204.8050	10.6 ± 0.9	12,670	− 34	0.997	27	0.5^x^
PFA	Perfluorooctanoic acid	PFOA	C_8_HF_15_O_2_	[M-H]^−^	412.9653	11.4 ± 0.8	2,222,303	310,401	0.999	8	0.04^y^
	Perfluoroctansulfonic acid	PFOSA	C_8_HF_17_O_3_S	[M-H]^−^	498.9291	11.6 ± 1.2	518,827	451,732	0.985	8	0.4^y^
HMSA	Trifluormethansulfonic acid	TFMSA	CF_3_SO_3_H	[M-H]^−^	148.9526	8.4 ± 0.5	10,222,591	1,945,661	0.998	13	0.03^y^
	Chloromethanesulfonic acid	MCMSA	CH_2_ClSO_3_H	[M-H]^−^	128.9419	8.7 ± 1.1	608,458	134,760	0.998	23	0.2^x^
	Bromomethanesulfonic acid	MBMSA	CH_2_BrSO_3_H	[M-H]^−^	172.8914	8.9 ± 0.7	420,783	105,773	0.998	22	0.2^x^
	Dichlormethanesulfonic acid	DCMSA	CHCl_2_SO_3_H	[M-H]^−^	162.9029	9.2 ± 0.8	1,387,925	233,245	0.997	17	0.2^x^
	Bromochloromethanesulfonic acid	BCMSA	CHBrClSO_3_H	[M-H]^−^	206.8524	9.4 ± 0.8	157,879	75,567	0.993	29	0.2^x^

Quantification of the targeted analytes by CE-MS was performed by measurements of seven water samples in triplicates and application of the external calibration curve obtained by triplicate measurements of standard solutions at the same time (randomly). The results for all target analytes in the drinking water samples are given in Table 2.

**Table 2:** Determined concentrations of HAAs, PFAs, and HMSAs by CE-MS in micrograms per liter in water samples (WS). The production plant is indicated by the number, a neutralization by thiosulfate by “T,” and an additional chlorination step by “C”

Group	Compound	Abbreviation	Sum formula	Ion	*m*/*z*	Sample name with measured concentration [μg/L ± stdev]						
						WS 1	WS 1 T	WS 2	WS 2 T	WS 3 T	WS 4	WS 4 C
HAA	Bromochloro acetic acid	BCAA	C_2_H_2_BrClO_2_	[M-COOH]^−^	126.8944	0.2 ± 0.1	0.3 ± 0.03	0.2 ± 0.1	0.4 ± 0.1	–	–	0.2 ± 0.1
	Trichloro acetic acid	TCAA	C_2_HCl_3_O_2_	[M-COOH]^−^	116.9060	0.4 ± 0.2	1.0 ± 0.03	0.3 ± 0.1	0.4 ± 0.2	0.5 ± 0.1	0.2 ± 0.1	0.2 ± 0.1
	Dichloro acetic acid	DCAA	C_2_H_2_Cl_2_O_2_	[M-H]^−^	126.9348	3.8 ± 1.1	6.3 ± 0.2	0.5 ± 0.2	1.8 ± 0.4	0.1 ± 0.1	0.2 ± 0.1	0.2 ± 0.1
	Dibromo acetic acid	DBAA	C_2_H_2_Br_2_O_2_	[M-COOH]^−^	170.8439	–	0.12 ± 0.1	–	–	–	< LOQ	–
	Dibromochloro acetic acid	DBCAA	C_2_HBr_2_ClO_2_	[M-COOH]^−^	204.8049	–	–	–	–	–	–	–
PFA	Perfluorooctanoic acid	PFOA	C_8_HF_15_O_2_	[M-H]^−^	412.9653	0.1 ± 0.02	0.1 ± 0.03	0.1 ± 0.02	0.3 ± 0.2	0.8 ± 0.4	0.8 ± 0.4	0.6 ± 0.3
	Perfluoroctansulfonic acid	PFOSA	C_8_HF_17_O_3_S	[M-H]^−^	498.9291	0.4 ± 0.01	0.4 ± 0.01	0.33 ± 0.1	0.5 ± 0.1	0.8 ± 0.2	1.0 ± 0.9	0.7 ± 0.01
HMSA	Trifluormethansulfonic acid	TFMSA	CF_3_SO_3_H	[M-H]^−^	148.9526	0.1 ± 0.04	0.3 ± 0.01	0.1 ± 0.02	0.10	0.10	0.1 ± 0.1	0.2 ± 0.01
	Chloromethanesulfonic acid	MCMSA	CH_2_ClSO_3_H	[M-H]^−^	128.9419	< LOQ	< LOQ	< LOQ	< LOQ	< LOQ	0.3 ± 0.03	< LOQ
	Bromomethanesulfonic acid	MBMSA	CH_2_BrSO_3_H	[M-H]^−^	172.8914	–	–	< LOQ	< LOQ	–	< LOQ	< LOQ
	Dichlormethanesulfonic acid	DCMSA	CHCl_2_SO_3_H	[M-H]^−^	162.9029	1.6 ± 0.7	2.2 ± 0.1	1.0 ± 0.4	0.7 ± 0.4	1.1 ± 0.1	> LOQ (2.3)	1.7 ± 0.1
	Bromochloromethanesulfonic acid	BCMSA	CHBrClSO_3_H	[M-H]^−^	206.8524	< LOQ	–	0.3 ± 0.1	< LOQ	< LOQ	> LOQ (3.6)	1.5 ± 0.1

### Halogenated acetic acids

**Fig. 3:**
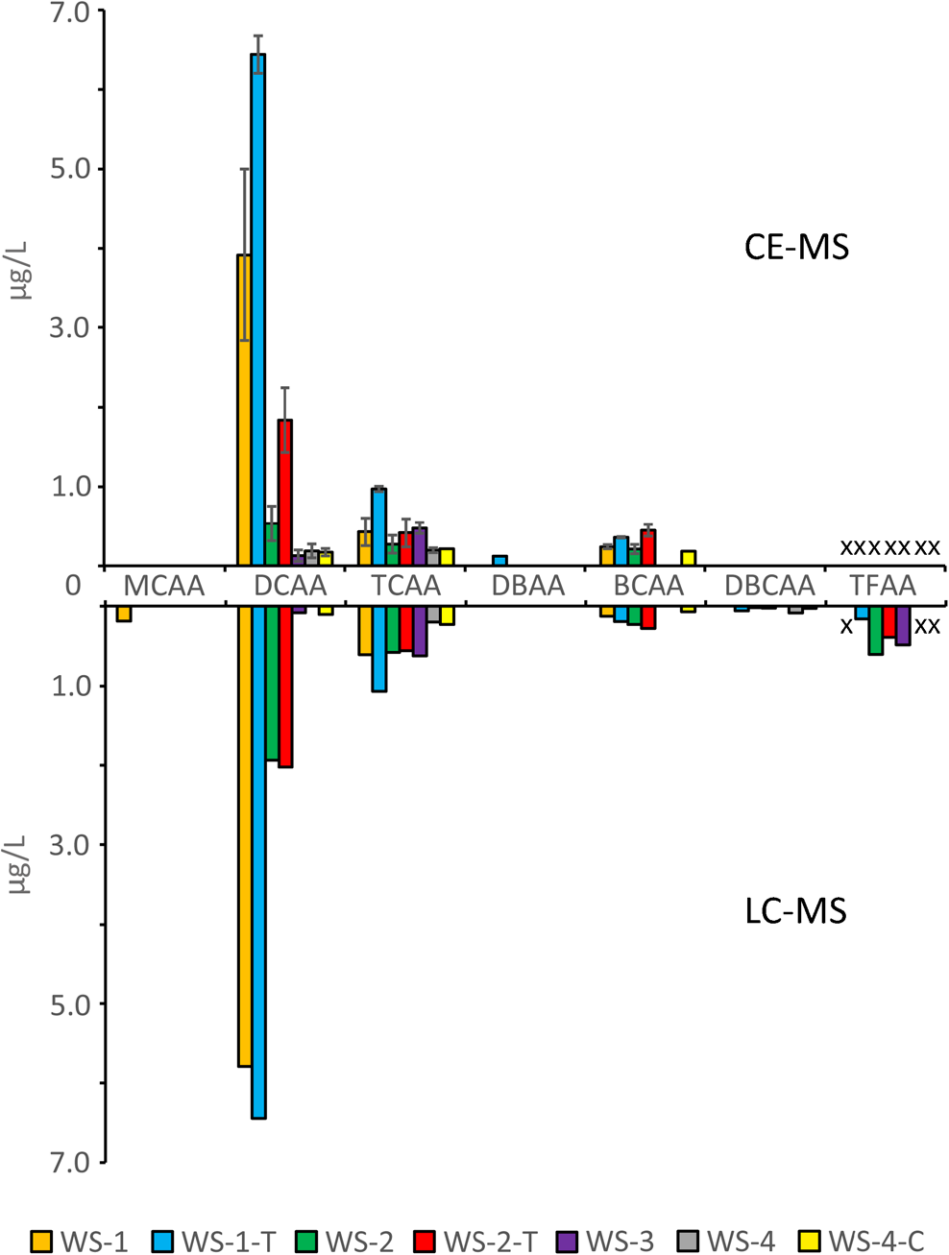
HAA concentrations determined by CE-MS and LC-MS in seven drinking water samples

BCAA, DCAA, and TCAA were found in most of the seven water samples in a range of 0.1 to 6.3 μg/L. The sum concentration of the HAAs is below 10 μg/L for all samples, which is the guideline level according to the Guidelines for Drinking-Water Quality of the WHO [26]. To verify the determined concentrations, the samples were analyzed and quantified by RPLC-MS/MS as an orthogonal method. The results of both techniques show similar concentrations (Fig. 3). With HPLC-MS, three additional HAAs could be quantified and were not found in the CE-MS measurements. On the other hand, by CE-MS, a low concentration of DBAA could be found additionally in one sample. TFAA could not be quantified for the CE-MS measurements due to high background signals, probably caused by contamination from other methods used in the laboratory. Additionally, subsequent LC-MS analysis revealed a contamination of three samples (marked by “x” in Fig. 3) and a quantification was not reasonable. The TFAA concentrations of the remaining four samples are in a plausible range for drinking water. The results for all drinking water samples are summarized in Table 2.

### PFOS and PFOA

The migration velocity of PFOs and PFOA is significantly slower compared to the HAAs which might be related to the larger molecular size and differences in pka value. Both analytes show a broader peak shape than other analytes, also due to longer analysis time but probably because of capillary wall interactions or a different stacking behavior. An example for the detected peaks can be found in Fig. 2. PFOS and PFOA were found in all DWPP samples from 0.1 to 0.7 μg/L, according to the calibration data. Since these findings are much higher than typical concentrations found in drinking water [27], a contamination of the samples cannot be excluded. However, blank values and laboratory tap water samples did not show any signals of PFOS or PFOA above LOQ. Thus, further measurements are needed to evaluate these findings and to determine matrix effects possibly influencing signal intensity in the presented CE-ESI-MS approach.

### Halomethanesulfonic acids

**Fig. 4:**
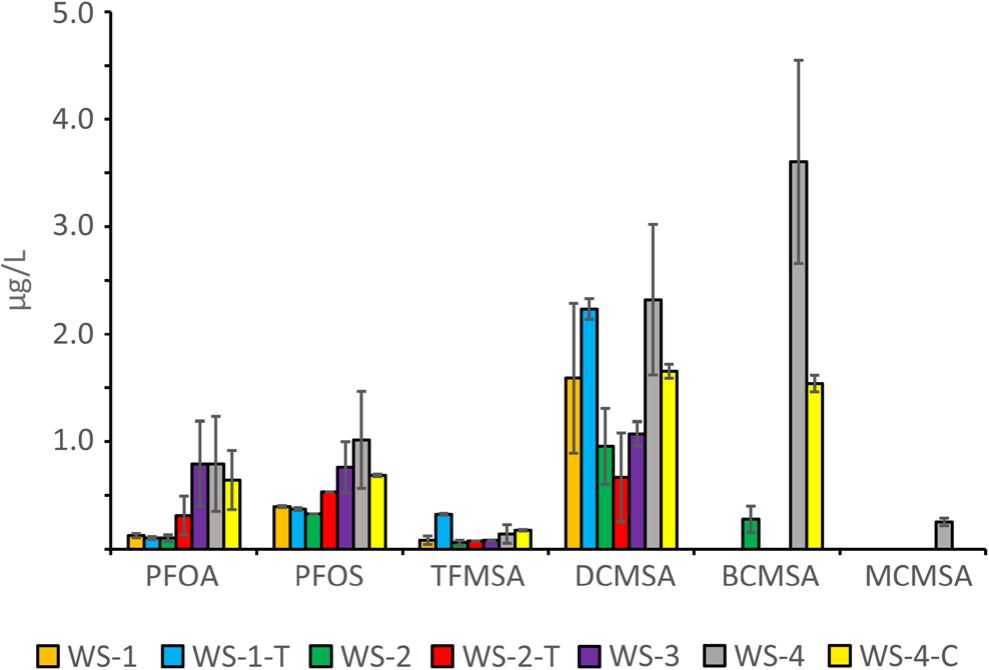
Determined concentrations of PFOA, PFOS, and four HMSAs found in seven drinking water samples

After data evaluation for the determination of HAAs, PFAs, and TFMSA, the CE-MS data were reviewed and scanned for additional HMSAs which might have been formed during the disinfection. Several sharp signals with increasing migration times close to TFMSA were found, some with characteristic isotopic patterns of ^81^Br and ^37^Cl. The group was baseline separated with six second baseline peak widths and migrated faster than the HAAs, which is related to a higher degree of dissociation due to negative pka values. An example of the separation in drinking water can be found in Fig. 2. The findings were confirmed in subsequent CE-MS and CE-MS/MS analyses with standards. To determine the concentration levels, calibration curves were recorded with four points from 2.0 to 0.2 μg/L with triple determination in ultrapure water with an acceptable linearity (*R*^2^ 0.993–0.998) and LOQs in the range of 0.03 to < 0.2 μg/L (cp. Table 1). However, the standard deviations were not optimal for exact quantification and the determined concentrations should be considered as semi-quantitative, nevertheless certainly being improved when internal standards will be used. TFMSA and DCMSA could be detected in all seven drinking water samples; however, integrated signal areas of MCMSA and MBMSA were mostly below the lowest calibration point. In one sample, the signal of BCMSA was higher than the calibration range but estimated to be around 3.5 μg/L for DCMSA and BCMSA, while TFMSA was generally below 0.3 μg/L. The quantitative results can be found in Table 2 and Fig. 4 and are similar to Zahn et al. [10]. The sum of all HMSAs is below 10 μg/L in all samples.

### Further suspects and non-target screening

The datasets were further screened for other micropollutants. The search could be narrowed to molecules with a low pka value, since the method is selective to molecules with a (partial) negative charge at pH 2.2. The data were searched by means of accurate mass (± 2.5 ppm) for over 20 suspects which included some halogenated and non-halogenated sulfonates and sulfates such as the anions of methanesulfonic acid, pentafluoroethanesulfonic acid, sulfamic acid, or ethyl sulfate but also the artificial sweeteners acesulfame and cyclamate and inorganic anions as halides, halogenates, sulfate, or phosphate. An exemplary electropherogram is shown in Fig. 2. The full list of these suspects with peak intensities and proposed compound with sum formula deviated from MS1 data is presented in Table S1 and Fig. S1 in the Electronic Supplementary Material (ESM). Some traces show double peaks in the same accuracy window, illustrating the need for further confirmation (migration time, MS-MS data) of these tentatively detected molecules. Nevertheless, a relative high confidence of these rather small molecules can be assumed based on the resolution of 30,000 and the mass accuracy (± 2.5 ppm) [28]. While the organic suspects are distributed over the whole migration time range, some of the inorganic suspects migrate faster and in broad bands which indicates that the stacking mechanism does not apply in their case, which makes the simultaneous quantification difficult. However, quantification with good peak shape could be performed by injecting a lower sample volume. Anyway, the observed separation of highly concentrated salts from the organic anions is beneficial for quantitative determination of these organic micropollutants since they do not interfere with their ionization. This is especially important for samples with a more complex matrix such as wastewater. However, maximum injection volumes are restricted for such samples with high conductivity, and thus, sensitivity of the CE-MS method is expected to be reduced.

A preliminary non-targeted approach for the data evaluation was tested for selected samples to explore the potential for finding additional features. The raw data of the drinking water samples were processed with MZmine 2 [29] against injections of calibration standards solved in ultrapure water to extract aligned feature lists. An absolute amount of over 5000 features was found, of which around 4000 were unique to the drinking water samples. Even with strict filtration criteria, over 300 features remained unique for the water samples. The filtration included removal of (a) features found in less than three of three injections, (b) features with peak intensities with a standard deviation larger than 25%, and (c) features which were found both in samples and blank injections. This number of features indicates the large amount of potential contaminants which could be determined in water samples by CE-MS in general.

In the field of metabolomics, the use of CE-MS for non-targeted screening is already widely accepted and the strength of CE for the separation of highly polar and ionic substances as a complementary technique to RPLC was evaluated for many applications and matrices [22, 30]. Even nanoflow interfacing techniques were already applied and non-targeted software solutions were developed to address the drawbacks of CE concerning migration time shifts by converting the time axis into effective mobility [31]. The here-presented results demonstrate the ability for the separation and high sensitivity detection of analytes of concern in drinking water. In context of the widely accepted use in the metabolomics community, CE-MS could be a helpful and complementary tool for the discovery of unknown and quantitation of identified PMOCS in various water samples.

## Conclusions

The here-presented work demonstrates the potential of CE-MS for the analysis of anionic micropollutants (expected to belong mostly to the group of PMOCs) in drinking water analysis. The ability for separation and quantification was proven with a range of analytes of concern like HAAs, PFAs, and HMSAs. Due to the selectivity of the method, it is possible to analyze various ions which are negatively charged under acidic conditions (pH ~ 2.2) as a complementary technique to more traditional chromatography. The absence of sample preparation offers the possibility to screen a multitude of analytes relevant in water analysis and even to discover unknowns, demonstrated here by the screening of organic and inorganic suspects and preliminary non-targeted screening detecting about 300 features. This screening approach is of interest, especially since it is expected that many ionic micropollutants are not discovered yet. The quantification limits reached by a simple large-volume injection and the use of a nanoflow sheath liquid interface are sufficient for the determination of pollutants in the sub-microgram-per-liter range. The method sensitivity could be improved by offline preconcentration techniques, commonly used in LC-MS approaches to reach LOQs in the nanogram per liter range. Also, the separation of inorganic ions from analytes of interest was achieved which allows their trace quantification as well as it is expected to cause less ion suppression in ESI, possibly faced by other techniques. To cover other substance classes such as weak acids or cationic contaminants, different modes of CE-MS can be applied. All these methods will promote wider use of CE-MS in environmental and water analysis, in conjunction with further advancement of nanoESI interfaces with respect to automation, ease of use, and robustness. Then, widely accepted and standardized analysis protocols as well as appropriate data evaluation workflows for non-targeted approaches by CE-MS should be developed, with possible adaptations from related fields such as untargeted metabolomics.

## Electronic supplementary material

ESM 1 (PDF 615 kb)

## References

[1] Reemtsma T (2016). Mind the gap: persistent and mobile organic compounds-water contaminants that slip through. Environ Sci Technol.

[2] Richardson SD (2018). Water analysis: emerging contaminants and current issues. Anal Chem.

[3] Schmidt TC (2018). Recent trends in water analysis triggering future monitoring of organic micropollutants. Anal Bioanal Chem.

[4] Scheurer M (2017). Small, mobile, persistent: trifluoroacetate in the water cycle - overlooked sources, pathways, and consequences for drinking water supply. Water Res.

[5] Montes R (2019). Determination of persistent and mobile organic contaminants (PMOCs) in water by mixed-mode liquid chromatography-tandem mass spectrometry. Anal Chem.

[6] Montes R (2019). Determination of persistent and mobile organic contaminants (PMOCs) in water by mixed-mode liquid chromatography-tandem mass spectrometry. Anal Chem.

[7] Schulze S, Zahn D, Montes R, Rodil R, Quintana JB, Knepper TP, Reemtsma T, Berger U (2019). Occurrence of emerging persistent and mobile organic contaminants in European water samples. Water Res.

[8] Yang C. Analysis of glyphosate and AMPA in environmental water by ion chromatography electrospray tandem mass spectrometry (IC-ESI-MS/MS). Thermo Fischer Application Note 2019 No. 491. https://assets.thermofisher.com/TFS-Assets/CMD/Application-Notes/AN-491-Glyphosate-AMPA-IC-ESI-MS-AN-63068.pdf.

[9] Zahn D (2016). Halogenated methanesulfonic acids: a new class of organic micropollutants in the water cycle. Water Res.

[10] Zahn D (2019). Halomethanesulfonic acids-a new class of polar disinfection byproducts: standard synthesis, occurrence, and indirect assessment of mitigation options. Environ Sci Technol.

[11] García A (2017). Capillary electrophoresis mass spectrometry as a tool for untargeted metabolomics. Bioanalysis.

[12] Stolz A (2019). Recent advances in capillary electrophoresis-mass spectrometry: instrumentation, methodology and applications. Electrophoresis.

[13] Gaspar A (2019). Determination of chlorine species by capillary electrophoresis - mass spectrometry. Electrophoresis.

[14] Medrano LC (2019). Solid-phase extraction and large-volume sample stacking-capillary electrophoresis for determination of artificial sweeteners in water samples. Food Anal Methods.

[15] Baciu T (2016). Capillary electrophoresis combined in-line with solid-phase extraction using magnetic particles as new adsorbents for the determination of drugs of abuse in human urine. Electrophoresis.

[16] Breadmore MC (2017). Recent advances in enhancing the sensitivity of electrophoresis and electrochromatography in capillaries and microchips (2014-2016). Electrophoresis.

[17] Ahrer W (1999). Determination of haloacetic acids by the combination of non-aqueous capillary electrophoresis and mass spectrometry. Fresenius J Anal Chem.

[18] Zhang H (2011). Pressure-assisted electrokinetic injection for on-line enrichment in capillary electrophoresis-mass spectrometry: a sensitive method for measurement of ten haloacetic acids in drinking water. Anal Chim Acta.

[19] Hu S (2018). Simultaneous determination of iodinated haloacetic acids and aromatic iodinated disinfection byproducts in waters with a new SPE-HPLC-MS/MS method. Chemosphere.

[20] Knob R (2012). On-line preconcentration of perfluorooctanoic acid and perfluorooctanesulfonic acid by nonaqueous capillary electrophoresis. Electrophoresis.

[21] Sun L (2015). Third-generation electrokinetically pumped sheath-flow nanospray interface with improved stability and sensitivity for automated capillary zone electrophoresis-mass spectrometry analysis of complex proteome digests. J Proteome Res.

[22] van Mever M (2019). CE-MS for anionic metabolic profiling: an overview of methodological developments. Electrophoresis.

[23] Höcker O (2018). Characterization of a nanoflow sheath liquid interface and comparison to a sheath liquid and a sheathless porous-tip interface for CE-ESI-MS in positive and negative ionization. Anal Bioanal Chem.

[24] Meng L (2010). Trace determination of nine haloacetic acids in drinking water by liquid chromatography-electrospray tandem mass spectrometry. J Chromatogr A.

[25] Bayless W (2008). Biodegradation of six haloacetic acids in drinking water. J Water Health.

[26] Organization WH (2011). Guidelines for drinking-water quality.

[27] Cordner A (2019). Guideline levels for PFOA and PFOS in drinking water: the role of scientific uncertainty, risk assessment decisions, and social factors. J Expo Anal Environ Epidemiol.

[28] Krauss M (2010). LC-high resolution MS in environmental analysis: from target screening to the identification of unknowns. Anal Bioanal Chem.

[29] Pluskal T (2010). MZmine 2: modular framework for processing, visualizing, and analyzing mass spectrometry-based molecular profile data. BMC Bioinformatics.

[30] Ramautar R (2019). CE-MS for metabolomics: developments and applications in the period 2016-2018. Electrophoresis.

[31] González-Ruiz V (2018). ROMANCE: a new software tool to improve data robustness and feature identification in CE-MS metabolomics. Electrophoresis.

