# Supplementary material for: Enrichment-free analysis of anionic micropollutants in the sub-ppb range in drinking water by capillary electrophoresis-high resolution mass spectrometry
Source: Anal Bioanal Chem. 2020 Mar 9;412(20):4857–65. doi: 10.1007/s00216-020-02525-8 (PMC7334245; doi:10.1007/s00216-020-02525-8)
Supplement: Supplementary file 1 — (PDF 615 kb) [file 216_2020_2525_MOESM1_ESM.pdf]

Analytical and Bioanalytical Chemistry

Electronic Supplementary Material

Enrichment-free analysis of anionic micropollutants in the sub-ppb range in drinking water by capillary electrophoresis-high resolution mass spectrometry

Oliver Höcker, Tobias Bader, Torsten C. Schmidt, Wolfgang Schulz, Christian Neusüß

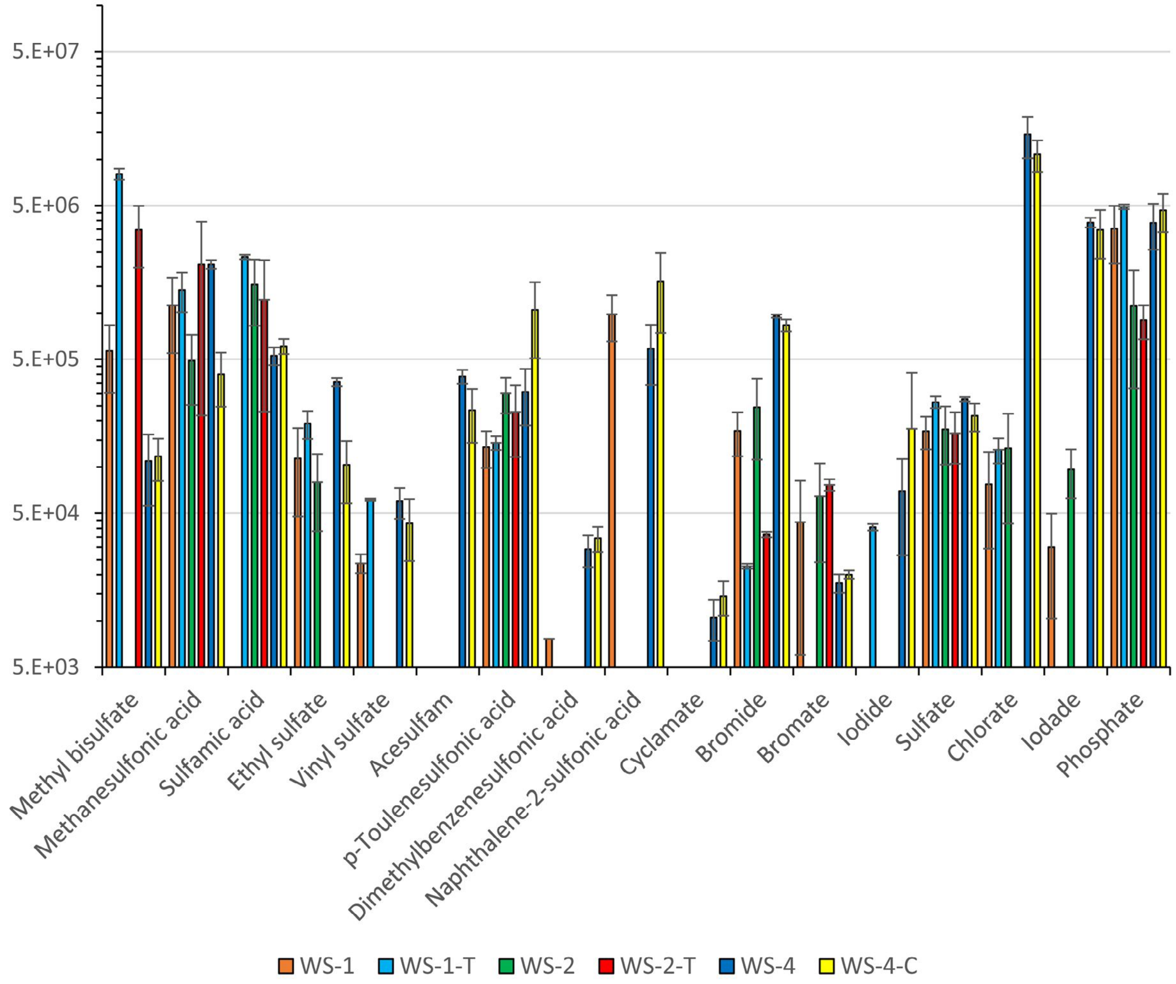

Fig. S1 Presentation of data from Table S1 of intensities in logarithmic scale of additional analytes found in the water sampels

Table S1 Organic and inorganic suspects that were screened in water samples. Only compounds found in all three replicates are listed. Sum formulas were deviated from exact mass on MS1 level

Group	Compound	Sum formula	Ion	Theoretical suspect m/z	Experimental m/z	m/z deviation [ppm]	Sample name and measured intensity [counts]					
							WS-1	WS-1-T	WS-2	WS-2-T	WS-4	WS-4-C
Organic suspects	Methyl bisulfate	CH_4O_4S	[M-H]-	110.9758	110.9755	2.1	6.E+05	8.E+06	-	3.E+06	1.E+05	1.E+05
	Methanesulfonic acid	CH_4SO_3	[M-H]-	94.9808	94.9806	2.4	1.E+06	1.E+06	5.E+05	2.E+06	2.E+06	4.E+05
	Sulfamic acid	H_3NSO_3	[M-H]-	95.9761	95.9759	2.4	-	2.E+06	2.E+06	1.E+06	5.E+05	6.E+05
	Ethyl sulfate	$C_2H_6O_4S$	[M-H]-	124.9914	124.9911	2.2	1.E+05	2.E+05	8.E+04	-	4.E+05	1.E+05
	Vinyl sulfate	$C_2H_4O_4S$	[M-H]-	122.9758	122.9755	2.2	2.E+04	6.E+04	-	-	6.E+04	4.E+04
	Acesulfam	$C_4H_5NO_4S$	[M-H]-	161.9867	161.9864	1.4	-	-	-	-	4.E+05	2.E+05
	p-Toulenesulfonic acid	$C_7H_8O_3S$	[M-H]-	171.0121	171.0121	0.6	1.E+05	1.E+05	3.E+05	2.E+05	3.E+05	1.E+06
	Dimethylbenzenesulfonic acid	$C_8H_{10}O_3S$	[M-H]-	185.0278	185.0277	0.4	8.E+03	-	-	-	3.E+04	3.E+04
	Naphthalene-2-sulfonic acid	$C_{10}H_8O_3S$	[M-H]-	207.0121	207.0118	1.7	1.E+06	-	-	-	6.E+05	2.E+06
	Cyclamate	$C_6H_{13}NO_3S$	[M-H]-	178.0543	178.0541	1.2	-	-	-	-	1.E+04	1.E+04
Inorganic suspects	Bromide	Br	[M]-	78.9189	78.9187	2.4	2.E+05	2.E+04	2.E+05	4.E+04	1.E+06	8.E+05
	Bromate	BrO_3	[M]-	126.9036	126.9041	4.1	4.E+04	-	6.E+04	8.E+04	2.E+04	2.E+04
	Iodide	I	[M]-	126.9050	126.9045	3.9	-	4.E+04	-	-	7.E+04	2.E+05
	Sulfate	SO_4	[M]-	82.9541	82.9540	2.4	2.E+05	3.E+05	2.E+05	2.E+05	3.E+05	2.E+05
	Chlorate	ClO_3	[M]-	95.9523	95.9525	2.0	8.E+04	1.E+05	1.E+05	-	1.E+07	1.E+07
	Iodade	IO_3	[M]-	174.8898	174.8896	0.7	3.E+04	-	1.E+05	-	4.E+06	3.E+06
	Phosphate	PO_3	[M]-	78.9591	78.9588	2.6	4.E+06	5.E+06	1.E+06	9.E+05	4.E+06	5.E+06
Screened suspects	Pentafluoroethanesulfonic acid	$C_2HF_5O_3S$	[M-H]-	198.9494	not found							
	Pentafluoropropionic acid	$C_3HF_5O_2$	[M-H]-	162.9824	not found							
	Heptafluoropropanesulfonic acid	$C_3HF_7O_3S$	[M-H]-	248.9462	not found							
	Perfluorobutanesulfonic acid	$C_4HF_9O_3S$	[M-H]-	298.9430	not found							
	Salicylic acid	$C_7H_6O_3$	[M-H]-	138.0322	not found							
